# Antioxidant Blueberry Anthocyanins Induce Vasodilation via PI3K/Akt Signaling Pathway in High-Glucose-Induced Human Umbilical Vein Endothelial Cells

**DOI:** 10.3390/ijms21051575

**Published:** 2020-02-25

**Authors:** Wuyang Huang, Ruth Paulina Hutabarat, Zhi Chai, Tiesong Zheng, Weimin Zhang, Dajing Li

**Affiliations:** 1 Institute of Agro-Product Processing & Jiangsu Key Laboratory for Horticultural Crop Genetic Improvement, Jiangsu Academy of Agricultural Sciences, Nanjing 210014, China; wuyanghuang@hotmail.com (W.H.); paulina.ruth84@ymail.com (R.P.H.); sophia_chai@163.com (Z.C.); 2 School of Food and Biological Engineering, Jiangsu University, Zhenjiang 212013, China; 3 Department of Food Science and Nutrition, Jinling College, Nanjing Normal University, Nanjing 210097, China; tieszheng@sina.com; 4 College of Food Science, Hainan University, Hainan 570228, China

**Keywords:** anthocyanin, antioxidant, blueberry, hypotensive, malvidin, vasodilatory

## Abstract

Blueberries are rich in antioxidant anthocyanins. The hypotensive effects of blueberry anthocyanins in endothelial cells was investigated here. Pretreatment with blueberry anthocyanin extract, malvidin, malvidin-3-glucoside, and malvidin-3-galactoside significantly ameliorated high-glucose-induced damage by enhancing endogenous antioxidant superoxide dismutase (SOD) and heme oxygenase-1 (HO-1), lowering reactive oxygen species (ROS) generation and NADPH oxidase isoform 4 (NOX4) expression, and increasing the cell vitalities. They also effectively induced a vasodilatory effect by increasing the vasodilator nitric oxide (NO) and its promoters endothelial NO synthase (eNOS) and peroxisome proliferator-activated receptor-γ (PPARγ) levels as well as by decreasing the vasoconstrictor angiotensin-converting enzyme (ACE), xanthine oxidase-1 (XO-1), and low-density lipoprotein (LDL) levels. The activation of phosphoinositide 3-kinase (PI3K)/Akt signaling pathway and the breakdown of protein kinase C zeta (PKCζ) pathway were involved in the bioactivities. The results indicated blueberry anthocyanins protected endothelial function against high-glucose (HG) injury via antioxidant and vasodilatory mechanisms, which could be promising molecules as a hypotensive nutraceutical for diabetes patients.

## 1. Introduction

Cardiovascular disease (CVD) is the leading cause of death in the world, with 80–90% of cardiovascular disease patients having hypertension [1]. The World Health Organization estimated that 17.6 million people died from cardiovascular disease in 2016, representing 44% of all global deaths. Diabetes is a typical risk factor that promotes cardiovascular diseases, especially hypertension [2]. According to the American Diabetes Association reports from 1999 to 2012, 71% of type 2 diabetes mellitus adult patients had hypertension [3]. Hyperglycemia is known to be the main contributing factor in the progression of the disease, which triggers pathological metabolic and biochemical changes that damage the cells [4]. Diabetes may increase blood pressure by reducing the blood vessels’ ability to stretch and, thus, aggravate endothelial injuries [5]. Vascular glycoxidative stress is an essential benefactor to endothelial dysfunctions in which it inhibits the production of nitric oxide (NO), a powerful vasodilator in the vascular system [6]. Therefore, diabetes and vascular glycoxidative stress aggravate vasoconstriction and induce the progression of hypertension [7].

Epidemiologic studies indicate the enhancement of antioxidant status is associated with a blood-pressure-lowering effect, since oxidative stress is a major cause of reduced endothelial NO availability [8]. Antioxidants can improve endothelial function by directly scavenging free radicals, reducing nicotinamide adenine dinucleotide phosphate (NADPH) oxidase, and increasing extracellular superoxide dismutase (SOD) activity, thus promoting endothelial NO availability and lower blood pressure [9]. Therefore, food-derived antioxidants exhibit antihypertensive effects and benefit for the prevention and treatment of cardiovascular disease. The Dietary Approaches to Stop Hypertension (DASH) eating plan recommends supplementation with vegetables and fruits, which are rich in antioxidant phenolics, including anthocyanins [10].

Several berries have been proved to have the hypotensive effects that can relax the blood vessels and lower the blood pressure via the regulation of vascular endothelium [11,12]. It might be because berry fruits contain an abundant amount of anthocyanins [13]. Anthocyanins are identified as nature’s most efficacious antioxidants that can successfully suppress free radicals and, thus, can prevent diabetes, hypertension, and other pathological diseases associated with oxidative stress [14]. Compared with other berry fruits, such as the strawberry, raspberry, blackberry, and cranberry, blueberry has more anthocyanidins [15]. Różańska and Regulska-Ilow found that malvidin was the most content/quantity anthocyanidin found in blueberry, followed by delphinidin, petunidin, peonidin, and cyaniding [16]. In our previous study, malvidin was also confirmed as the largest anthocyanidin found in Brightwell rabbiteye blueberry (*Vaccinium ashei*) of Nanjing, mostly as malvidin-3-glucoside (Mv-3-glc) and malvidin-3-galactoside (Mv-3-gal) [17]. We tried to investigate the different bioactivities of blueberry anthocyanin extract as well as its major constituents Mv-3-glc and Mv-3-gal and found they could improve endothelial function by protecting against oxidative stress [18,19]. However, only a few reports are about the vasodilation activities of anthocyanin extract from blueberry fruits [20]. The hypotensive effects of blueberry anthocyanins in human endothelial cells are also still not clear. In the present study, the protective effects of blueberry anthocyanin extract, as well as malvidin, malvidin-3-glucoside, malvidin-3-galactoside, in high-glucose-stimulated human umbilical vein endothelial cells (HUVECs) were investigated to propose a functional mechanism for their role as hypotensive agents.

## 2. Results

### 2.1. Protective Effects of Blueberry Anthocyanins on High-Glucose (HG)-Induced Cytotoxicity in HUVECs

**Figure 1:**
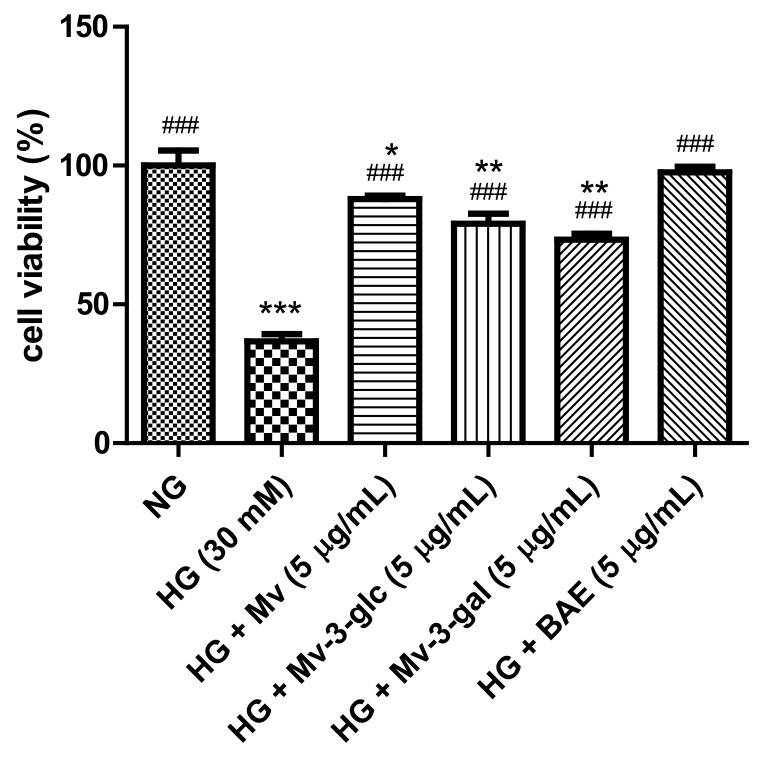
Effects of malvidin (Mv), malvidin-3-glucoside (Mv-3-glc), malvidin-3-galactoside (Mv-3-gal), and blueberry anthocyanin extract (BAE) on cell viability in human umbilical vein endothelial cells (HUVECs) exposed to high glucose for 24 h. Bars represent mean values ± SD (*n* = 3). *, **, and *** indicate *p* < 0.05, *p* < 0.01, and *p* < 0.001, respectively, compared with the control (normal-glucose group, NG); ### indicates *p* < 0.001 compared with the high-glucose (HG) model.

High glucose causes multiple biological effects and contributes to cellular cytotoxicity [21]. Figure 1 shows the effects of high glucose and blueberry anthocyanins on the cell viability in HUVECs. In this study, high-glucose stimulation for 24 h significantly decreased the cell viability (HG, 30 mM: 36.67 ± 2.71% vs. NG, 5.5 mM: 100 ± 5.39%; *p* < 0.001). Pretreatment with 5 µg/mL of malvidin (Mv), malvidin-3-glucoside (Mv-3-glc), malvidin-3-galactoside (Mv-3-gal), and blueberry anthocyanin extract (BAE) could significantly ameliorate the cell viability of HG-induced HUVECs to 87.94 ± 1.21%, 79.10 ± 3.61%, 73.37 ± 2.10%, and 97.60 ± 1.91%, respectively (all *p* < 0.001 vs. HG group). Blueberry anthocyanin extract improved HUVEC’s cell viability nearly the same as the control. Malvidin possessed a significantly better protective effect on the cell viability than its derivatives Mv-3-glc and Mv-3-gal (*p* < 0.05 and *p* < 0.001 vs. Mv group, respectively). The cell viability of Mv-3-glc was slightly higher than that of Mv-3-gal, but the difference was not significant (*p* > 0.05).

### 2.2. Protective Effects of Blueberry Anthocyanins against HG-Induced ROS Levels in HUVECs

**Figure 2:**
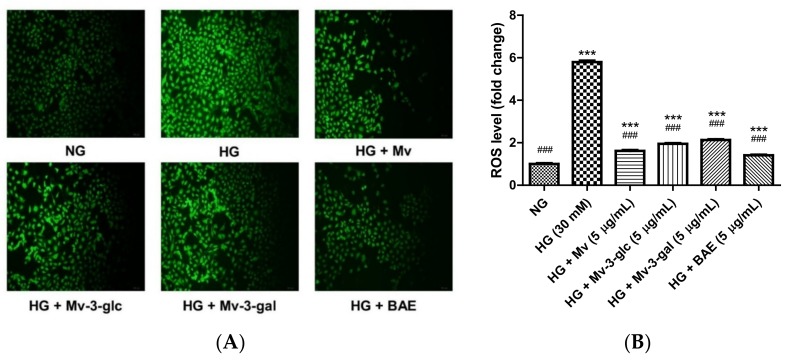
Effects of Mv, Mv-3-glc, Mv-3-gal, and BAE on reactive oxygen species (ROS) level in HUVECs exposed to high glucose for 24 h: (**A**) The fluorescence intensity (**B**) ROS fold change. A representative set of images from three independent experiments is shown. All images presented are in ×200 magnification. Bars represent mean values ± SD (*n* = 3). *** indicates *p* < 0.001 compared with the control NG; ### indicates *p* < 0.001 compared with the HG model.

A dichloro-dihydro-fluorescein diacetate (DCFH-DA) detection kit was used to assess the ROS level in HUVECs. The total fluorescence intensity of cells in each well was noted. The fluorescence intensity in the cells of normal-glucose treatment was low. After stimulation with high glucose, the fluorescence intensity of HUVECs was significantly enhanced, which indicated that the level of reactive oxygen species (ROS) was increased. However, fluorescence intensity was decreased after treatment with Mv, Mv-3-glc, Mv-3-gal, and BAE (Figure 2A). The ROS level of HUVECs exposed to HG for 24 h was 5.79 ± 0.07 fold that of the control (*p* < 0.001 vs. NG group). Pretreatment with 5 μg/mL Mv, Mv-3-glc, Mv-3-gal, and BAE significantly inhibited ROS formation by about 87.12 ± 0.79%, 80.20 ± 0.67%, 76.48 ± 0.75%, and 91.38 ± 0.48%, respectively (all *p* < 0.001 vs. Mv group, Figure 2B). Similarly, BAE showed the strongest antioxidant effect by decreasing the most ROS level. Malvidin reduced ROS production more than its derivatives, while Mv-3-glc decreased more ROS levels than Mv-3-gal (*p* < 0.01).

### 2.3. Effects of Blueberry Anthocyanins by Upregulating Antioxidant SOD and HO-1 Levels in HG-Induced HUVEC Supernatant

**Figure 3:**
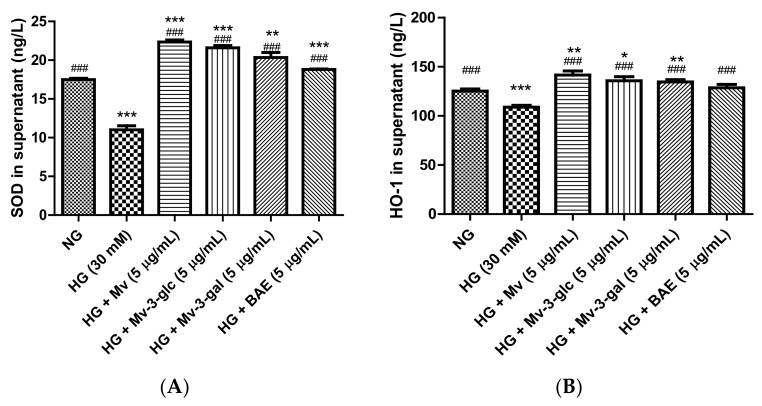
Effects of Mv, Mv-3-glc, Mv-3-gal, and BAE on antioxidant (**A**) superoxide dismutase (SOD) and (**B**) heme oxygenase-1 (HO-1) production released into the supernatant of HUVECs exposed to high glucose for 24 h. Bars represent mean values ± SD (*n* = 3). *, **, and *** indicate *p* < 0.05, *p* < 0.01, and *p* < 0.001, respectively, compared with the control NG; ### indicates *p* < 0.001 compared with the HG model.

Superoxide Dismutase (SOD) and Heme Oxygenase-1 (HO-1) are enzymes that act as important endogenous antioxidant-defense systems mainly present in endothelial cells that ameliorate endothelial functions by scavenging the ROS and preventing the formation of reactive free radicals [22,23]. In the present study, SOD and HO-1 protein expression levels were high in unstimulated cells and were strongly downregulated when exposed to high glucose (*p* < 0.001). Pretreatment with Mv, Mv-3-glc, Mv-3-gal, and BAE could effectively upregulate the SOD and HO-1 protein expression levels (Figure 3). The SOD and HO-1 levels in Mv-, Mv-3-glc-, Mv-3-gal-, and BAE-pretreated HUVEC supernatant were 2.03 and 1.30 fold, 1.96 and 1.25 fold, 1.85 and 1.24 fold, and 1.71 and 1.18 fold that of those in HG-induced HUVEC supernatant, respectively (all *p* < 0.001 vs. HG group). The inhibitory rates of Mv, Mv-3-glc, Mv-3-gal, and BAE against SOD and HO-1 decrease were all above 100%, whose levels were even more than those in the control (SOD: all *p* < 0.001 vs. NG group; HO-1: *p* < 0.05 vs. NG group except BAE). Malvidin still showed a better capability to increase the downregulation of SOD and HO-1 expression levels than its derivatives Mv-3-glc and Mv-3-gal; however, BAE possessed the lowest expression levels of antioxidant enzymes SOD and HO-1 in HUVEC supernatant.

### 2.4. Vasodilatory Effects of Blueberry Anthocyanins by Upregulating NO and eNOS Levels in HG-Induced HUVEC Supernatant

**Figure 4:**
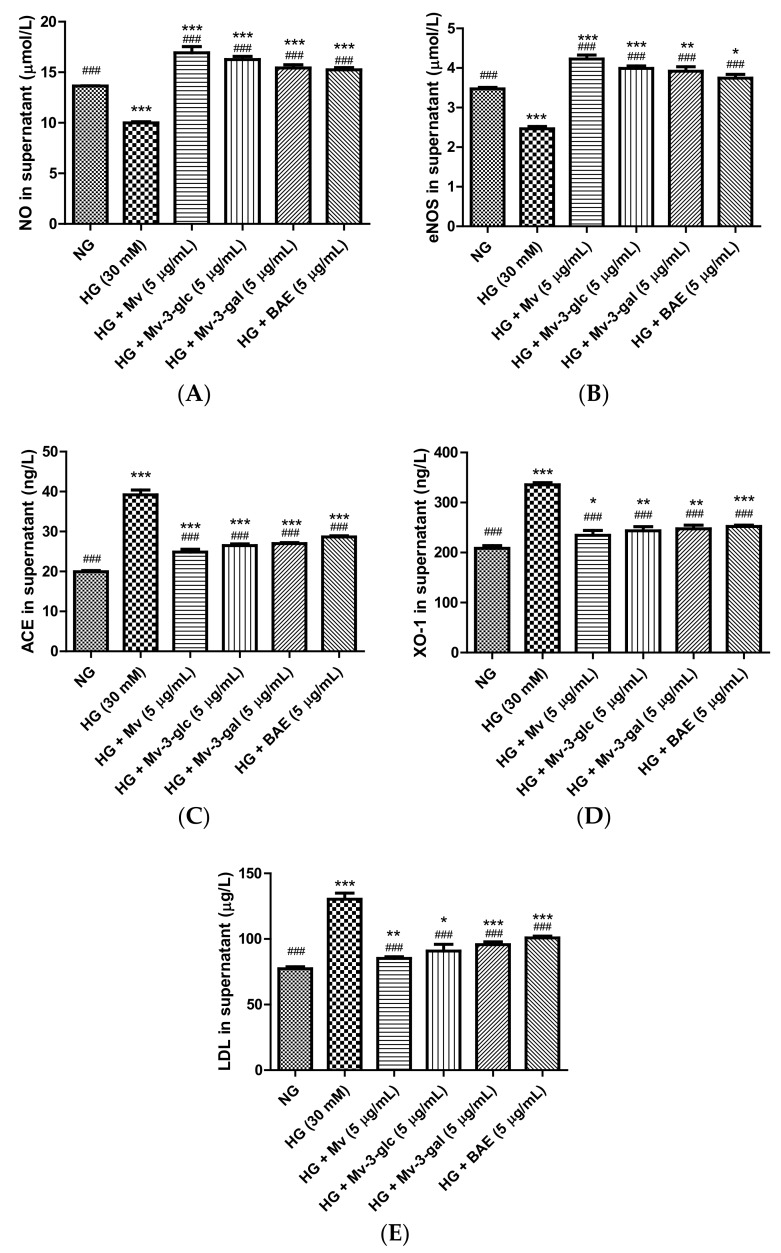
Vasodilatory effects of Mv, Mv-3-glc, Mv-3-gal, and BAE by regulating nitric oxide (NO) (**A**), endothelial nitric oxide synthase (eNOS) (**B**), angiotensin-converting enzyme (ACE) (**C**), xanthine oxidase-1 (XO-1) (**D**), and low-density lipoprotein (LDL) (**E**) production released into the supernatant of HUVECs exposed to high glucose for 24 h. Bars represent mean values ± SD (*n* = 3). *, **, and *** indicate *p* < 0.05, *p* < 0.01, and *p* < 0.001, respectively, compared with the control NG; ### indicates *p* < 0.001 compared with the HG model.

Nitric oxide (NO) is a powerful vasodilator produced by endothelial NO synthase (eNOS) in the vascular endothelium [24]. In this study, extracellular NO and eNOS levels were high in the unstimulated cell supernatant and were strongly downregulated when exposed to high glucose (*p* < 0.001). Pretreatment with Mv, Mv-3-glc, Mv-3-gal, and BAE could restore these downregulations of NO and eNOS (*p* < 0.001). Their inhibitory rates against NO and eNOS decrease were all above 100%, with 1.69 and 1.72 fold, 1.63 and 1.62 fold, 1.54 and 1.59 fold, and 1.53 and 1.52 fold that of HG-NO level and HG-eNOS, respectively (all *p* < 0.001 vs. HG group, Figure 4A,B). Malvidin showed the strongest vasodilatory ability to increase NO and eNOS levels in HUVEC supernatant, followed by Mv-3-glc, Mv-3-gal, and BAE (*p* < 0.05).

### 2.5. Vasodilatory Effects of Blueberry Anthocyanins by Downregulating ACE, XO-1, and LDL Levels in HG-Induced HUVEC Supernatant

Angiotensin-converting enzyme (ACE) indirectly elevates blood pressure by converting the hormone angiotensin I to the progressive vasoconstrictor angiotensin II [25]. Xanthine oxidase-1 (XO-1), a major source of superoxide, is implicated in endothelial dysfunction partly due to the rapid inactivation of vasodilator NO production [26]. Circulated low-density lipoprotein (LDL) particles in the blood can permeate through the inner lining of the blood vessel and be trapped in the arterial walls, in which the exaggerated aggregation of LDL particles can form lipid-rich plaque and worsen the endothelial injury [27]. In the present study, the effects of blueberry anthocyanins on extracellular ACE, XO-1, and LDL levels in HG-induced HUVEC supernatant were also evaluated using enzyme-linked immunosorbent assay (ELISA) analysis.

Protein expression levels of ACE, XO-1, and LDL were low in the control cells and were strongly upregulated to 1.97 ± 0.06, 1.61 ± 0.02, and 1.69 ± 0.06 folds that of the control when exposed to high glucose (*p* < 0.001 vs. NG group). Pretreatment with Mv, Mv-3-glc, Mv-3-gal, and BAE significantly reduced the up-regulation of ACE, XO-1, and LDL expression levels in high glucose-induced HUVEC supernatant (all *p* < 0.001 vs. HG group). Their inhibitory rates were 74.36 ± 3.56%, 65.89 ± 2.14%, 63.34 ± 1.30%, and 54.70 ± 1.48%, for HG-induced ACE, 79.54 ± 7.53%, 72.49 ± 6.64%, 69.39 ± 5.79%, and 65.72 ± 2.28% for XO-1, and 85.12 ± 2.50%, 74.54 ± 9.66%, 65.26 ± 3.98%, and 55.53 ± 2.49% for LDL respectively (Figure 4C–E). Similar to the results of NO and eNOS, malvidin showed the strongest vasodilatory ability to decrease ACE, XO-1, and LDL levels in HUVEC supernatant, followed by Mv-3-glc, Mv-3-gal, and BAE.

### 2.6. Antioxidant Effects of Blueberry Anthocyanins by Downregulating NOX4 Levels in HG-Induced HUVECs

**Figure 5:**
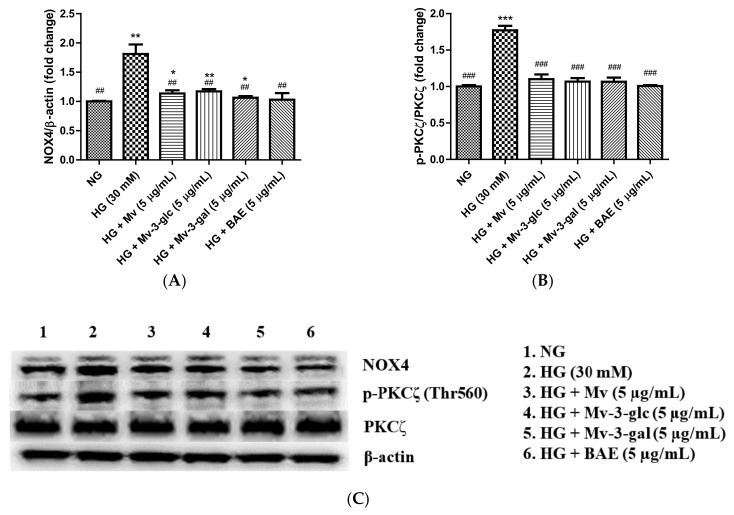
Downregulatory effects of Mv, Mv-3-glc, Mv-3-gal, and BAE on (**A**) NADPH oxidase isoform 4 (NOX4) and (**B**) p-PKCζ/PKCζ levels in HUVECs exposed to high glucose for 24 h. (**C**) Representative western blot bands are shown. Bars represent mean values ± SD (*n* = 3). *, **, and *** indicate *p* < 0.05, *p* < 0.01, and *p* < 0.001, respectively, compared with the control NG; ## and ### indicate *p* < 0.01 and *p* < 0.001, respectively, compared with the HG model.

NADPH oxidase isoform 4 (NOX4) induces oxidative stress by generating a high level of H_2_O_2_ and superoxide anion (O_2_^•^^−^) [28]. Western blotting analysis showed that intracellular protein expression levels of NOX4 were low in the control cells and were strongly upregulated when exposed to high glucose (*p* < 0.01). Pretreatment with Mv, Mv-3-glc, Mv-3-gal, and BAE showed a similar ability to reduce the upregulation of NOX4 expression levels in high-glucose-induced HUVECs significantly. The inhibitory rates of Mv, Mv-3-glc, Mv-3-gal, and BAE on HG-induced NOX4 were 83.20 ± 6.65%, 78.71 ± 4.85%, 92.19 ± 3.49%, and 96.27 ± 13.91%, respectively (all *p* < 0.01 vs. HG group, Figure 5A). Representative western blot bands are shown in Figure 5C.

### 2.7. Vasodilatory Effects of Blueberry Anthocyanins by Upregulating eNOS and PPARγ Levels in HG-Induced HUVECs

**Figure 6:**
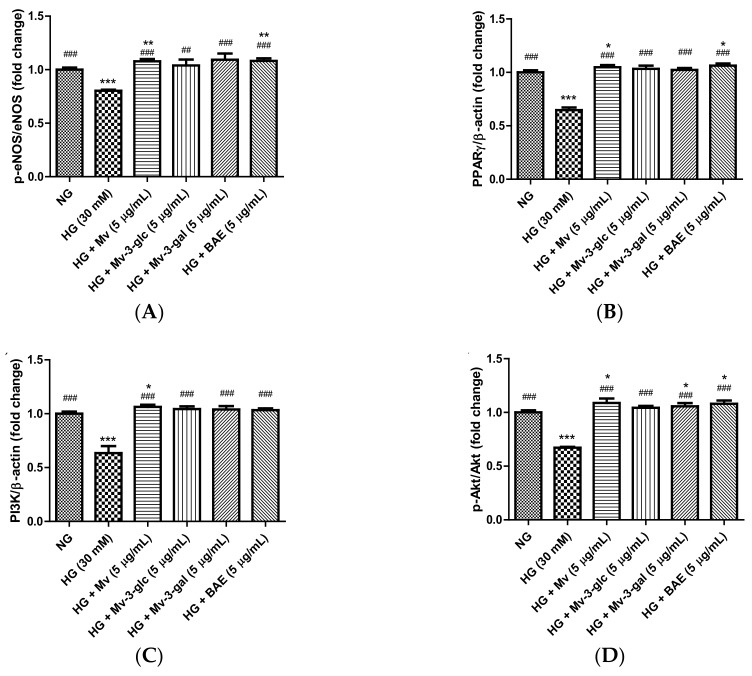

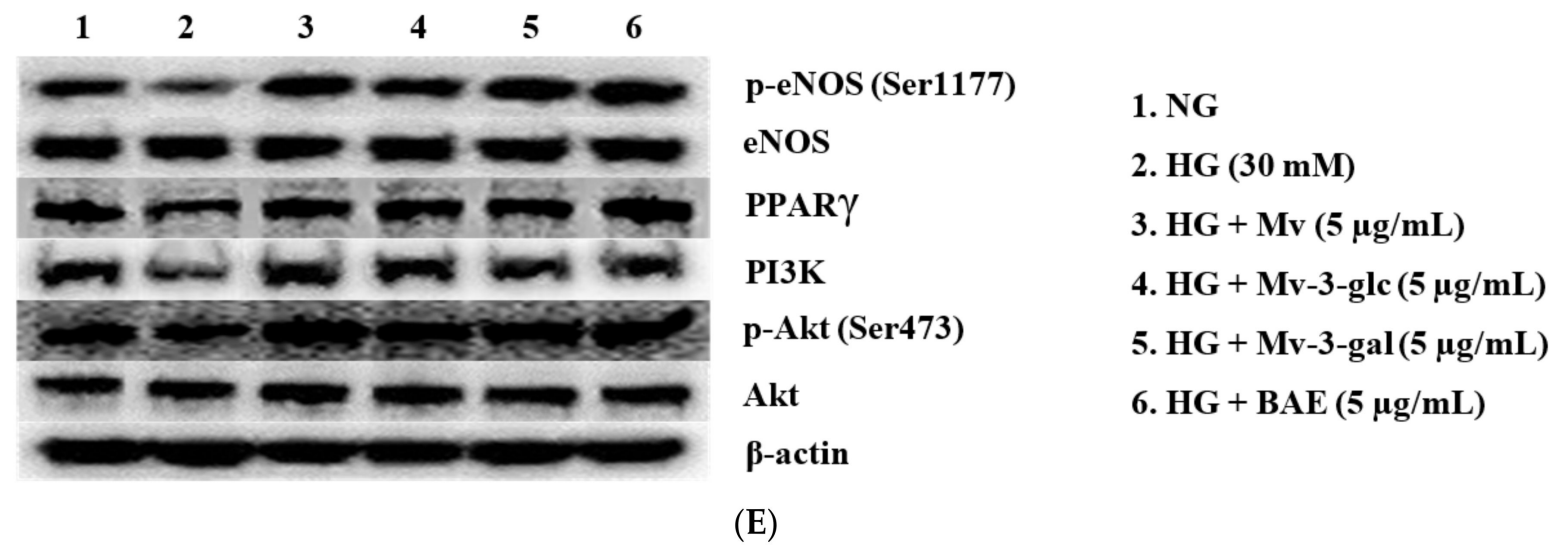
Upregulatory effects of Mv, Mv-3-glc, Mv-3-gal, and BAE on (**A**) p-eNOS/eNOS, (**B**) peroxisome proliferator-activated receptor-γ (PPARγ), (**C**) phosphoinositide 3-kinase (PI3K), and (**D**) p-Akt/Akt levels in HUVECs exposed to high glucose for 24 h. (**E**) Representative western blot bands are shown. Bars represent mean values ± SD (*n* = 3). *, **, and *** indicate *p* < 0.05, *p* < 0.01, and *p* < 0.001, respectively, compared with the control NG; ## and ### indicate *p* < 0.01 and *p* < 0.001, respectively, compared with the HG model.

Enzyme eNOS and peroxisome proliferator-activated receptor-γ (PPARγ) agonists promote NO generation in endothelial cells, and eNOS is activated through the phosphorylation at Ser1177 [29,30]. In the present study, the effects of blueberry anthocyanins on intracellular p-eNOS/eNOS and PPARγ levels in HG-induced HUVECs were evaluated using western blotting. Similar to extracellular eNOS level in the supernatant, pretreatment with Mv, Mv-3-glc, Mv-3-gal, and BAE could restore the downregulations of active p-eNOS protein expression levels in the cells by HG stimulation. The p-eNOS/eNOS ratio significantly decreased to 0.80 ± 0.01 fold that of the control when exposed to high glucose (*p* < 0.001 vs. NG group), while Mv, Mv-3-glc, Mv-3-gal, and BAE increased the p-eNOS/eNOS ratios to 1.34 ± 0.03, 1.29 ± 0.07, 1.36 ± 0.07, and 1.35 ± 0.03 fold that of the HG group, respectively (Mv-3-glc: *p* < 0.01; the others *p* < 0.001 vs. HG group). Protein expression levels of PPARγ were high in unstimulated cells and were strongly downregulated when exposed to high glucose. The inhibitory rates of Mv, Mv-3-glc, Mv-3-gal, and BAE on HG-induced PPARγ decrease were also above 100%, i.e., 113.45 ± 5.75%, 109.34 ± 8.57%, 106.67 ± 4.89%, and 117.78 ± 5.61%, respectively (all *p* < 0.001 vs. HG group). There was no significant difference in the p-eNOS/eNOS and PPARγ change among Mv, Mv-3-glc, Mv-3-gal, and BAE groups (*p* > 0.05; Figure 6A,B). Representative western blot bands are shown in Figure 6E.

### 2.8. Vasodilatory Effects of Blueberry Anthocyanins by PI3K/Akt Signaling Pathway and PKC Signaling Pathway in HG-Induced HUVECs

The serine/threonine kinase Akt is activated by phosphoinositide 3-kinase (PI3K), which plays a critical role in regulating diverse cellular functions. Many experiments have validated that the PI3K/Akt-eNOS signaling pathway is involved in the vasorelaxant effect [31]. The effects of blueberry anthocyanins on PI3K/Akt signaling pathway in HUVECs were evaluated using western blotting. Protein expression levels of PI3K and pAkt were high in the unstimulated cells and were strongly downregulated when exposed to high glucose (both *p* < 0.001 vs. NG group). Pretreatment with Mv, Mv-3-glc, Mv-3-gal, and BAE could restore all these downregulations of PI3K and pAkt. The enhancement rates of Mv, Mv-3-glc, Mv-3-gal, and BAE were 1.17 fold, 1.12 fold, 1.11 fold, and 1.09 fold for PI3K expression and 1.27 fold, 1.13 fold, 1.17 fold, and 1.25 fold for p-Akt/Akt, respectively (all *p* < 0.001 vs. HG group; Figure 6C,D,E).

PKCζ activation decreases eNOS protein stability and induces endothelial cell apoptosis [32]. Protein expression levels of p-PKCζ/PKCζ were low in the control cells and were strongly upregulated to 1.77 ± 0.06 fold that of the control when exposed to high glucose (*p* < 0.001 vs. NG group). The inhibitory rates of Mv, Mv-3-glc, Mv-3-gal, and BAE on p-PKCζ/PKCζ were 86.83 ± 8.37%, 91.28 ± 6.28%, 91.46 ± 7.36%, and 99.07 ± 1.61%, respectively (all *p* < 0.001 vs. HG group; Figure 5B,C). There was no significant difference in the PI3K, p-Akt/Akt, and p-PKCζ/PKCζ change among Mv, Mv-3-glc, Mv-3-gal, and BAE groups (*p* > 0.05).

## 3. Discussion

Endothelium, located in the interface of the vessel wall and the bloodstream, plays a protective role in blood vessels, which regulates cardiovascular functions, including blood circulation, vascular tone, and platelet aggregation, thereby maintaining vascular homeostasis and blood pressure [4]. Vascular oxidative stress is determined as the state of excessive ROS, such as superoxide anion and hydrogen peroxide, that overwhelmes the antioxidant system in the endothelium [33]. Nitric oxide (NO) is a powerful vasodilator that inhibites the constriction of blood vessels triggered by hyperaggregation of thrombocytes and, thus, widenes the blood vessels and smoothes the flow of arterial blood [6]. Superoxide anion reacts rapidly with NO in the vasculature and yields peroxynitrite that depletes the bioactivity of NO. Hydrogen peroxide also yields peroxynitrite when it reacts with nitrite [34]. Therefore, ROS produced in vascular systems contribute to endothelial injury by reducing the NO production, consequently causing vasoconstriction and hypertension [7]. High glucose generates ROS in the vascular system, in which free radicals formed by ROS decrease the blood flow and induce hypertension [35]. In this study, the upraised level of ROS after 24 h of high-glucose stimulation explained that high glucose raised the intracellular level of vascular oxidative stress in endothelial cells and disturbed the vascular cellular homeostasis. The decrease of cell viability after 24 h of high-glucose stimulation explained that high glucose caused the degradation of glucose consumption and uptake and led to endothelial injuries and apoptosis. These might be the reasons why most diabetes patients have hypertension.

Anthocyanins are nature’s most efficacious antioxidants, and blueberries contain the highest anthocyanin contents among the tested fruits and vegetables [36]. A number of epidemiological and pharmacological studies confirm that blueberry anthocyanins possess a wonderful antioxidant capacity in vitro and in vivo [37,38]. Our previous studies have confirmed that malvidin-3-glucoside and malvidin-3-galactoside are major efficient anthocyanins in blueberries and display pronounced antioxidant properties in endothelial cells [39,40]. In this study, pretreatment with blueberry anthocyanin extract, malvidin, and its derivatives has been proved to decrease ROS generation and ameliorate cell viability in vascular system. Blueberry anthocyanin extract showed the strongest antioxidant ability to protect HUVECs from vascular glycoxidative stress attack, followed by malvidin, malvidin-3-glucoside, and malvidin-3-galactoside. The different glycosylation pattern of anthocyanins can impact their antioxidant activity, either enhancing or diminishing it, because the glycosylation effects have relied on the environment when the oxidation occurs [41]. Miguel reported that the glycosylation pattern of anthocyanins was less effective than the aglycone pattern in reducing free hydroxyls and metal chelation sites [14]. Malvidin-3-glucose possess better bioavailability than malvidin-3-galactoside. This might be due to its greater efficacy of binding to the organic anion membrane carrier, bilitranslocase [42]. The crude blueberry anthocyanin extract more pronouncedly protected cell viability against ROS oxidative stress than malvidin and its derivatives might because some other anthocyanins in the blueberry extract also contributed to the antioxidnt capacity.

Additionally, endothelial cells play a role in regulating the oxidant/antioxidant balance by producing reactive oxygen species (ROS) as well as antioxidants such as SOD and HO-1 [43]. HO-1 and SOD were ROS-detoxifying enzymes with large antioxidant capacity that mainly present in endothelial cells [44]. In this study, blueberry anthocyanin extract, malvidin, and its derivatives have been successfully proved to increase HO-1 and SOD expression levels in high-glucose-induced HUVECs. On the other hand, antioxidants can improve endothelial function by reducing NADPH oxidase (a superoxide producing enzyme); our western blotting analysis confirmed that blueberry anthocyanin extract, malvidin, and its derivatives could decrease high-glucose-induced NOX4 expression levels in HUVECs. Thus, blueberry anthocyanins could improve endothelial functions by scavenging the ROS and preventing reactive free radical generation, thus smoothening the blood flow in endothelial cells. These results indicated that blueberry anthocyanins have a powerful antioxidant ability to prevent endothelial cells from glycoxidative deterioration by decreasing ROS formation in vasculature. Therefore, it should be an effective nutraceutical applied to prevent endothelial dysfunction triggered by vascular glycoxidative stress, such as diabetes.

Nitric oxide (NO), as the endothelium-derived vasodilator, signals the surrounding smooth muscle to relax, thus increasing blood flow and decreasing blood pressure, which is biosynthesized endogenously from L-arginine, oxygen, and NADPH by NOS enzymes [34]. Phosphorylation is an important regulator of eNOS enzymatic activity in regulation of insulin sensitivity and energy metabolism, suggesting eNOS phosphorylation as a novel target for the treatment of diabetes [24]. In this study, blueberry anthocyanin extract, malvidin, and its derivatives have successfully been proved to increase NO and eNOS activation by the phosphorylation of eNOS at the Ser1177 active site in high-glucose-induced HUVECs. Our data suggest that the vasodilator mechanism of blueberry anthocyanins seems to be associated with NO production by endothelial NOS in the PI3K/Akt-dependent pathway. PI3K directly activates Akt/PKB via phosphorylation at two different sites, which are Thr308/309 at A-loop in the kinase domain and Ser473/474 at the carboxy-terminal region [45]. Protein kinase B (PKB), also known as Akt, contributes to angiogenesis by activating eNOS, which increases the production of NO [46]. In this study, blueberry anthocyanins have successfully been proved to increase PI3K activity and Akt activation by the phosphorylation at Ser473 active site in high-glucose-induced HUVECs. Another mechanism that might been proposed to explain vasodilation is promoting the PPARγ pathway. PPARγ agonists can downregulate the Wnt/beta-catenin pathway, which is involved in inflammation, endothelial dysfunction, the proliferation of vascular smooth muscle cells, and vascular calcification, therefore PPARγ agonists might be therapeutic targets for the treatment of atherosclerosis [47]. PPARγ agonist have been found to enhance NO availability in pre-hypertensive rats [30]. Here, blueberry anthocyanin extract, malvidin, and its derivatives acted as a good PPARγ agonist in high-glucose-induced HUVECs. Therefore, blueberry anthocyanins could effectively induce vasodilator NO production in human endothelial cells, dilate the blood vessel, and enhance the growth of new capillary blood vessels, consequently, decreasing the blood pressure.

Endothelial injuries contribute to the endothelial dysfunctions and induce the advancement of hypertension [48]. ACE, XO-1, NOX4, and PKCζ are enzymes mainly present in endothelial cells that aggravate the endothelial injuries. ACE produces the vasoconstrictor angiotensin II, thus ACE inhibitors are important antihypertensive drugs [25]. The ACE inhibitory activity of blueberry anthocyanin extract, malvidin, and its derivatives can be elucidated by the aptitude of flavonoid to chelate the metal ions such as iron or chopper with hydroxyl groups at 3, 5, 7, and 3′, 4′ positions at the active site of ACE [49]. XO-1 catalyzes the oxidation of xanthine to uric acid and hydrogen peroxidase, thus causing hyperuricemia, an abnormally upraised concentration of uric acid in the blood [50]. Uric acid acts as a pro-oxidant by yielding radicals in the reaction with other oxidants, and the formed radicals elevate the oxygenation of LDL, a group of lipoproteins which transports all fat molecules around the peripheral tissues, in which an oxidized LDL alters uric acid into oxidant [51,52]. This pro-oxidant effects of uric acids rapidly inactivate NO production in endothelial cells and augment the vascular oxidative harm [26]. The ability of blueberry anthocyanins to inhibit XO-1 activity is due to the replacement of OH in C-5 and C-7 positions with sugars, and the saturation and disfiguration of the double bond between C-2 and C-3 positions and B ring that is united by the unification to A and C rings [53]. 

NADPH oxidase isoform 4 (NOX4), a predominant isoform expressed in vascular cells, induces glycoxidative stress by transferring the electrons across biological membranes to an acceptor molecule, usually oxygen, thereby generating a high level of hydrogen peroxide (H_2_O_2_) and superoxide anion (O_2_^•^^−^) [27]. Inhibition of NOX reduces the scavenging of NO by superoxide anion; therefore, it increases the NO bioavailability and improves endothelial functions [54]. In this study, blueberry anthocyanin extract, malvidin, and its derivatives could effectively inhibit NOX-stimulated ROS production activity. Apocynin, a commercial NOX inhibitor, has a similar structure with a methylated B-ring of flavonoids [55]. Blueberry anthocyanins have great potential to be developed as commercial NOX inhibitor nutraceutical. PKCζ activation induces collective and directional migration of endothelial cells during angiogenesis, which attenuates the eNOS stability and induces endothelial cells apoptosis [32,56]. PKCζ is activated by phosphorylation at the Thr410 or Thr560 active site [57]. Blueberry anthocyanin extract, malvidin, and its derivatives here inhibited the phosphorylation of PKCζ at the Thr560 active site and, thus, could improve the generation of NO and new blood vessels in endothelial cells. Therefore, blueberry anthocyanins were proved in this study to enhance endothelial functions and improve vasodilatory effects.

## 4. Materials and Methods 

### 4.1. Materials and Chemicals

Blueberry anthocyanin extract (BAE) was obtained from rabbiteye blueberry fruits harvested by Fujiabian Orchard Picking (Lishui, Nanjing, China) [18]. Malvidin (Mv), malvidin-3-glucose (Mv-3-glc), malvidin-3-galactose (Mv-3-gal), and 3-4,5 dimethylthiazol-2,5 diphenyl tetrazolium bromide (MTT) were purchased from Sigma Aldrich (Shanghai, China). Bicinchoninic acid (BCA) protein assay kit were purchased from CoWin Biotechnology (Beijing, China). Dulbecco’s modified Eagle medium (DMEM), fetal bovine serum (FBS), penicillin–streptomycin were purchased from Gibco (Auckland, New Zealand). Dichloro-dihydro-fluorescein diacetate (DCFH-DA) detection kit was purchased from Beyotime Institute of Technology (Shanghai, China). AndyGene human superoxide dismutase (SOD), xanthine oxidase-1 (XO-1), heme oxygenase-1 (HO-1), low-density lipoprotein (LDL), nitric oxide (NO), endothelial nitric oxide synthase (eNOS), and angiotensin-converting enzyme (ACE) ELISA kits were purchased from Shanghai Bluegene Biotech (Shanghai, China). All chemical and reagents used were of analytical grade. 

### 4.2. Antibodies

Primary antibodies against eNOS, p-Akt (Ser473), phosphoinositide 3-kinase (PI3K) p110β, and protein kinase C zeta (PKCζ) were purchased from Cell Signaling Technology (Beverly, MA, USA). Primary antibodies against p-eNOS (SerS1177), Akt, p-PKCζ, and nicotinamide adenine dinucleotide phosphate oxidase NOX4 were purchased from Abcam (Cambridge, United Kingdom). Antibody against β-actin was purchased from Sigma Aldrich (St. Louis, MO, USA). Antibody against peroxisome proliferator-activated receptor-γ (PPARγ) was purchased from Nanjing Beidi Biomed Technology (Nanjing, China). Goat anti-mouse/rabbit IgG horseradish peroxidase (HRP)-conjugated secondary antibodies were purchased from Cell Signaling Technology (Beverly, MA, USA). Primary antibodies were used at 1:1000 dilutions. Secondary antibodies were used at 1:4000 dilutions.

### 4.3. Endothelial Cell Culture and Treatment

Human umbilical vein endothelial cells (HUVECs), derived from the well-differentiated endothelium of veins from the umbilical cord, are commonly utilized as a model system for the study of endothelial function [58]. HUVECs obtained from CoWin Biotechnology (Beijing, China) were cultured in DMEM containing normal glucose (NG, 5.5 mM) supplemented with 10% FBS and 1% penicillin–streptomycin and kept at 37 °C and 5% CO_2_ atmosphere in an incubator (Thermo Scientific, Waltham, MA, USA). HUVECs of third-to-fifth passage cells were used for all experiments after reaching 70–80% confluence. HUVECs were quiesced in a reduced serum medium for 4 h prior to the experiment. After pretreatment with the 5 µg/mL of Mv, Mv-3-glc, Mv-3-gal, or BAE for 24 h, the cells were exposed to high glucose (HG, 30 mM) to mimic hypertensive conditions. HUVECs with NG were used as the control. The supernatants were collected for ELISA analysis, while the cells were prepared for western blotting analysis.

### 4.4. Cell Viability Assay

The cell viability was determined by the MTT method [59]. After pretreatment with the 5 µg/mL of Mv, Mv-3-glc, Mv-3-gal, or BAE for 24 h and continuing with 5.5 or 30 mM glucose for 24 h, the cells were added with 10 μL 0.5% (5 mg/mL) of MTT and then continued to be cultured for 4 h. After removal of the MTT solution, the cell crystal was dissolved by adding 100 μL DMSO and shaking for 10 min slowly. The absorbance of 490 nm was measured on a StatFax-2100 microplate reader (Awareness Technology Inc., Plam, FL, USA). The cells were cultured only when normal glucose (5.5 mmol/L) was used as the control group. The blank group used the wells without cells. The cell viability was determined with the following formula:

(1) $$\mathrm{Cell}\;\mathrm{viability}\;(\%)\;=\;\frac{\mathrm{sample}\;\mathrm{group}\;\mathrm{OD}\;\mathrm{value}\;-\;\mathrm{blank}\;\mathrm{group}\;\mathrm{OD}\;\mathrm{value}}{\mathrm{control}\;\mathrm{group}\;\mathrm{OD}\;\mathrm{value}\;-\;\mathrm{blank}\;\mathrm{group}\;\mathrm{OD}\;\mathrm{value}}\times 100\%.$$

### 4.5. Reactive Oxygen Species (ROS) Assay

A dichloro-dihydro-fluorescein diacetate (DCFH-DA) detection kit was used to assess the ROS level in HUVECs. After pretreatment with the 5 µg/mL of Mv, Mv-3-glc, Mv-3-gal, or BAE for 24 h and continuing with 5.5 mM or 30 mM glucose for 24 h, cells were washed with PBS, and then 10 µM DCFH-DA was added to each well and reacted for 20 min at 37 °C, and the cells were washed thoroughly with PBS. A group of cells was visualized under an IX53 inverted fluorescent microscope (Olympus, Tokyo, Japan) with 530 nm emission and 485 nm excitation filters immediately. All images presented are in ×200 magnification. Another group of cells was collected after dissociation, and fluorescence was recorded by a Synergy H4 multi-mode microplate reader (BioTek Instruments Inc., Winooski, VT, USA). The total fluorescence intensity of cells in each well was noted, and ROS generation was measured as a fold of the control.

### 4.6. Enzyme-Linked Immunosorbent Assay (ELISA) Analysis

The extracellular protein level of NO, e-NOS, ACE, HO-1, SOD, XO-1, and LDL in the supernatants were quantified using ELISA kits. The total cell protein of the supernatant in each well was detected using a BCA protein assay kit. The absorbance of 450 nm was measured at 37 °C on a StatFax-2100 microplate reader (Awareness Technology Inc., Plam, FL, USA) to determine protein levels.

### 4.7. Western Blotting

The intracellular protein level of eNOS, p-eNOS (SerS1177), PI3K p110β, Akt, p-Akt (Ser473), PPARγ, NOX4, PKCζ, p-PKCζ was analyzed by western blotting performed on the HUVEC lysates. β-Actin was used as a loading control. Following a blocking step with 5% non-fat dry milk, the membrane was probed with a primary antibody. After a subsequent washing step, the membrane was incubated with a conjugated secondary antibody labeled with horseradish peroxidase (HRP). Then Pierce enhanced chemiluminescence (ECL) substrate kit, a chemiluminescence substrate for the HRP enzyme, was carefully applied to the blot, and the light emitted was captured by a FUSION Solo2 chemiluminescence image system (Vilber Lourmat, Marne La Vallée, France) with a charge coupled device (CCD) camera. The density of all bands was quantified using Bio Profile 1D++ software (Vilber Lourmat, Marne La Vallée, France). All data were expressed as a fold change to the control.

### 4.8. Statistical Analysis

All data presented are the mean value ± standard deviation (SD) of three independent experiments. Figures were obtained using GraphPad Prism Version 5 (GraphPad Software, Inc., CA, USA). One-way ANOVA or t-test was performed to determine statistical differences between different groups. Differences were considered significant with *p* < 0.05.

## 5. Conclusions

Blueberry anthocyanin extract as well as the major constituent malvidin and its derivatives Mv-3-glc, Mv-3-gal have been proved in this study as a potential hypotensive nutraceutical that could efficaciously ameliorate endothelial functions and improve vascular health in this study. High glucose induced great injury in human umbilical vein endothelial cells. Malvidin, Mv-3-glc, Mv-3-gal, and BAE all benefited cell growth of HUVECs with higher cell viability than the HG-stimulated group. They had great antioxidant effects by decreasing ROS levels and increasing enzyme activity of SOD and HO-1 in HUVECs. Downregulation of NOX4 expression might be one of their antioxidant mechanisms. Upregulation of NO, eNOS, and PPARγ levels might be another antioxidant mechanism for blueberry anthocyanins, which also contributed to their vasodilatory effects. In addition, malvidin, Mv-3-glc, Mv-3-gal, and BAE still also prevent endothelial-dysfunction-induced vasoconstriction by decreasing the expression levels of ACE, XO-1, and LDL in human endothelial cells. The PI3K/Akt signaling pathway, as well as the PKCζ pathway contributed to the blueberry anthocyanin-mediated antioxidant and vasodilatory activities in HUVECs. In general, malvidin exhibited better bioactivities than its derivatives Mv-3-glc and Mv-3-gal. Interestingly, the crude blueberry anthocyanin extract more pronouncedly protected cell viability against ROS oxidative stress than the pure anthocyanins; however, it had less or the same change of relative protein or enzyme expression in HG-induced HUVECs. The results indicated blueberry anthocyanins could be assumed as a potential hypotensive nutraceutical and thereby prevent cardiovascular diseases, such as hypertension caused by diabetes.
